# Expanding the Clinical and Mutational Spectrum of the *PLP1*-Related Hypomyelination of Early Myelinated Structures (HEMS)

**DOI:** 10.3390/brainsci11010093

**Published:** 2021-01-13

**Authors:** Francesco Nicita, Chiara Aiello, Gessica Vasco, Massimiliano Valeriani, Fabrizia Stregapede, Andrea Sancesario, Michela Armando, Enrico Bertini

**Affiliations:** 1 Unit of Neuromuscular and Neurodegenerative Diseases, Department of Neuroscience, IRCCS Bambino Gesù Children’s Hospital, 00146 Rome, Italy; chiara.aiello@opbg.net (C.A.); fabrizia.stregapede@opbg.net (F.S.); bertini@opbg.net (E.B.); 2 Laboratory of Molecular Medicine, Department of Neuroscience, IRCCS Bambino Gesù Children’s Hospital, 00146 Rome, Italy; 3 Unit of Neurorehabilitation, Department of Neurorehabilitation, IRCCS Bambino Gesù Children’s Hospital, 00050 Rome, Italy; gessica.vasco@opbg.net (G.V.); andrea.sancesario@opbg.net (A.S.); michela.armando@opbg.net (M.A.); 4 Unit of Neurology, Department of Neuroscience, IRCCS Bambino Gesù Children’s Hospital, 00165 Rome, Italy; valeriani@opbg.net; 5 Department of Sciences, University of Roma Tre, 00146 Rome, Italy; 6 Department Systems Medicine, University of Roma Tor Vergata, 00133 Rome, Italy

**Keywords:** HEMS, *PLP1*, DM20, exon 3B, intron 3, proteolipid protein 1, leukodystrophy, hypomyelinating, Pelizaeus-Merzbacher disease

## Abstract

The *PLP1* gene, located on chromosome Xq22, encodes the proteolipid protein 1 and its isoform DM20. Mutations in *PLP1* cause a spectrum of white matter disorders of variable severity. Here we report on four additional HEMS patients from three families harboring three novel *PLP1* mutations in exon 3B detected by targeted next-generation sequencing. Patients experienced psychomotor delay or nystagmus in the first year of age and then developed ataxic–spastic or ataxic syndrome, compatible with a phenotype of intermediate severity in the spectrum of *PLP1*-related disorders. Regression occurred at the beginning of the third decade of the eldest patient. Extrapyramidal involvement was rarely observed. Brain MRI confirmed the involvement of structures that physiologically myelinate early, although the pattern of abnormalities may differ depending on the age at which the study is performed. These new cases contribute to expanding the phenotypic and genotypic spectrum of HEMS. Additional studies, especially enriched by systematic functional evaluations and long-term follow-up, are welcome to better delineate the natural history of this rare hypomyelinating leukodystrophy.

## 1. Introduction

The *PLP1* gene, located on chromosome Xq22, encodes the proteolipid protein 1—the most abundant protein of myelin sheath in the central nervous system (CNS)—and its isoform DM20 that derives from alternative splicing due to an acceptor site within the exon 3, with subsequent production of a smaller protein that lacks the PLP1-specific domain encoded by amino acids 117–151 [1]. Expression of the two isoforms is tightly regulated in space and time, since DM20 is mainly expressed in the developing brain before the onset of myelination, whereas expression of PLP1 prevails during the myelination stage and after the process is completed as well. Mutations in *PLP1* alter myelin formation, and cause a spectrum of disorders with distinct genotype–phenotype correlations and variable severity (Table S1), most of them belonging to the group of hypomyelinating leukodystrophies. Leukodystrophies are genetic disorders primarily and predominantly affecting the white matter of the CNS with or without peripheral nervous system myelin involvement. Among the *PLP1*-related disorders, Pelizaeus-Merzbacher disease (PMD) is the more severe and best-known condition. Conversely, features of hypomyelination of early myelinated structures (HEMS) have been described in less than twenty cases [2]. Currently available data identify HEMS phenotype as relatively mild, and consistent with an ataxic-spastic syndrome, and preserved or mildly affected cognition [2].

However, as it rarely occurs and is a recently recognized phenotype, additional description of patients allows to increase awareness and improve counseling for patients and families.

In this paper, we present four unreported HEMS patients from three families harboring three novel *PLP1* mutations in exon 3B and describe their main clinical, genetic and neuroradiological data, to expand the phenotypic and genotypic spectrum of this rare hypomyelinating leukodystrophy.

## 2. Materials and Methods

Patients were identified from a cohort of 42 consecutive patients with a clinical and neuroradiological diagnosis of leukodystrophy collected from January 2017 to November 2020 at the Unit of Neuromuscular and Neurodegenerative Diseases of the Bambino Gesù Children’s Research Hospital and screened by means of targeted next-generation sequencing for leukodystrophy-causing genes.

Selected patients had a diagnosis of HEMS and were found to have a hemizygous mutation in the exon 3B or intron 3 of the *PLP1* gene. Accurate recording of age at onset and type of symptoms, neurological examination, brain magnetic resonance imaging (MRI) (if available) findings, and genetic data were retrospectively collected and analyzed.

Massively parallel sequencing was performed on index cases by a Nextera Custom Enrichment panel (Illumina, San Diego, CA, USA), including 76 previously described leukodystrophy-causing genes (list available upon request), according to manufacturers’ protocols. Variants were annotated by the HaplotypeCaller tool of GATK ver. 3.4 (Cambridge, MA, USA), and were annotated with ANNOVAR. Variants were filtered out to exclude those located in intronic regions and synonymous variants not predicted to affect splice sites, as well as non-synonymous variants with reported minor allele frequency (MAF) ≥0.01 in publicly available human variation resources (dbSNP146, 1000 Genomes, Exome Aggregation Consortium (ExAC), NHLBI Exome Sequencing Project (ESP), Exome Variant Server, and gnomAD). Pathogenicity of the identified variants was evaluated in accordance with the American College of Medical Genetics and Genomics (ACMG) guidelines [3]. Variants were annotated with reference to the canonical transcript NM_000533.4. Signed written informed consent for genetic analysis and for study participation was obtained from all patients or parents of enrolled individuals.

## 3. Results

### 3.1. Clinical Histories

**Figure 1:**
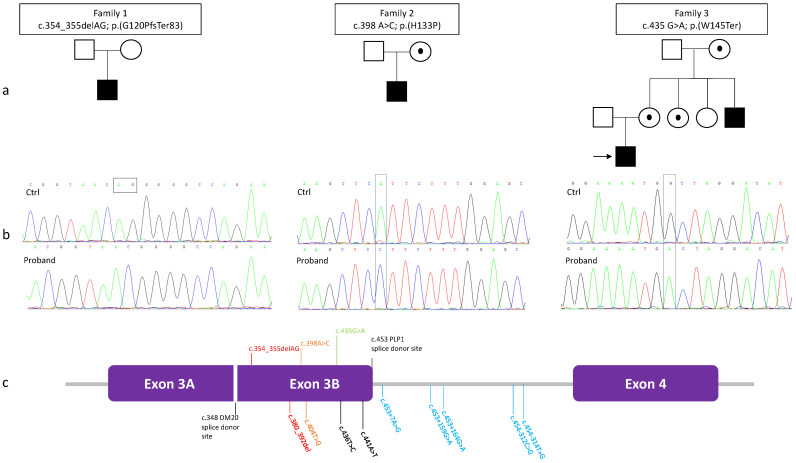
(**a**) Pedigrees of the three families: affected males are shown as black squares while female carriers are shown as white circles with black dots; proband in family 3 is indicated by a black arrow. (**b**) Electropherogram of sequencing analysis demonstrating the presence of a mutation in the proband and not in control. (**c**) Schematized close-up of exon 3-intron 3-exon 4 containing all the reported HEMS-causing mutations. Previously reported mutations are shown in the lower part, while the three novel variants discovered here are shown in the upper part of the figure. Color map indicates different types of variants: frameshift in red, missense in orange, silent in black, nonsense in green, intronic in light blue.

Data of four male patients from three families (Figure 1a, Table 1).

**Figure 2:**
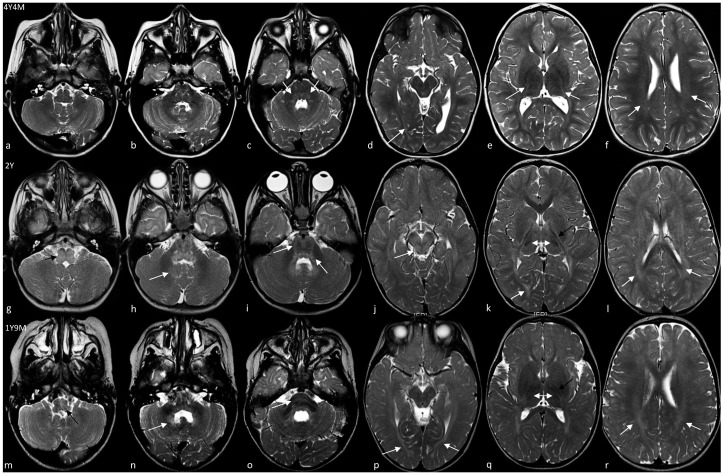
T2-weighted axial brain MRI of patient 1 (**a**–**f**), 2 (**g**–**l**), and 3 (**m**–**r**). In patient 1, medulla (**a**), dentate nuclei (**b**), and (**d**) midbrain are spared while mild hyperintensity is observed in dorsal pons (white arrows in **c**), optic radiations (white arrow in **d**), PLIC (white arrows in **e**) and periventricular white matter (white arrows in **f**). Patients 2 and 3 showed a more similar pattern of anomalies, with a high signal of the medulla (black arrow in g and **m**), hilus of the dentate nuclei (white arrow in **h** and **n**), pons (white arrow in **i** and **o**), optic radiations (white arrow in **k** and **p**) and periventricular white matter (white arrows in **l** and **r**). Please also note that the midbrain is hyperintense only in patient 2 (white arrow in **j**), that PLIC has a typical tram track appearance (black arrow in **k** and **q**), and that the dorsomedial thalami are hyperintense compared to the ventrolateral part (double white arrows in **k** and **q**).

Patient 1 is a 5.5-year-old boy with a history of psychomotor delay and hypotonia in the first year, followed by slow improvement over the years. He was born at term after uneventful pregnancy by cesarean delivery due to fetal macrosomia (i.e., weight greater than 4000 g regardless of baby’s gestational age). When he came to our attention, at the age of 4, neurological examination showed an ataxic gait with upper limbs tremor and dysarthria; osteotendinous reflexes were only mildly increased at lower limbs without further pyramidal signs. Brain MRI showed mild signs of hypomyelination (i.e., an increased signal in the T2/FLAIR images) with the involvement of pons, periventricular white matter, and posterior limb of internal capsulae (PLIC) (Figure 2a–f, Table 2) that remained unchanged one year later (data not shown). Nerve conduction study was normal. Visual evoked potential (VEP) showed bilateral increased latency. Brainstem auditory evoked potentials (BAEPs) detected upper pontine alterations. Currently, he is able to walk with little support and speak with simple sentences. No episodes of regression have been recorded.

Patient 2 is a 3 year 4 months old child born at term by cesarean section due to advanced maternal age. Pregnancy was uneventful, as well as delivery and neonatal period. Pendular nystagmus appeared at age 6 months and was followed by a delay in motor and language development. Brain MRI at the age of 2 showed signs of hypomyelination at the level of the medulla and pons, dentate nuclei, optic radiations, PLIC with tram track appearance, white matter of periventricular regions and corona radiate (Figure 2g–l, Table 2). At last evaluation, the child showed an ataxic–spastic syndrome, but was able to walk and run; nystagmus improved. He could speak with appropriate language for his age.

Patient 3 is an 8-year-old boy born at term by caesarean delivery for premature rupture of membranes with meconium-stained in amniotic fluid during labor. Apgar scores were 9-9 at 1′ and 5′ minutes, respectively. He started manifesting a psychomotor delay by the age of 9 months. At the age of 1 and a half years, the child could not sit unsupported, and a language delay was also evident. The neurological evaluation showed strabismus, axial hypotonia, and pyramidal hypertonia of lower limbs. Brain MRI revealed increased signal in the T2/FLAIR images with the involvement of medulla and pons, dentate nuclei, optic radiations, PLIC with tram track appearance, white matter of periventricular regions, and corona radiata (Figure 2m–r, Table 2) consistent with hypomyelination. Visual evoked potential revealed a bilateral reduction of amplitude and delayed latencies. Over the years, the child showed slow improvement in motor and cognitive function without evidence of neurodegeneration. A further brain MRI revealed some modifications of a hypomyelinating pattern (Table 2, Figure S1). At the last examination, at the age of 8, he manifested strabismus, drooling, head and trunk titubation, intentional tremor of upper limbs. Dyskinetic orofacial movements were also recorded, and therapy with tetrabenazine was prescribed. Pyramidal spasticity of lower limbs was evident and required focal treatment with botulin injection. Currently, he attends elementary school with a support teacher; he is able to sit with lateral support, to walk some steps with a walker, and to use a wheelchair for long distances. He has a moderate ability to push the wheelchair independently. The speech is impaired with dysarthria and words are difficult to understand most of the time. In the same family, the 46-year-old maternal uncle (patient 4) had a similar clinical history. Specifically, he was born at term with premature rupture of membranes and labor dystocia. At birth, he showed mild signs of hypoxic-ischemic encephalopathy but no further treatment was started at that time. Psychomotor delay and hypertonic syndrome were evident from the first year of life, but no investigations were carried out. He developed spastic ataxia over the years, and was able to walk with lateral support from the age of 2.5 to 20 years when he lost the ability to walk due to worsening of motor functions. At last examination, at the age of 46, he showed spastic tetraparesis with cerebellar signs, was wheelchair-bound and had no swallowing problems. His speech was unintelligible and he was completely dependent for activities of daily living.

### 3.2. Genetic Findings

Three novel pathogenic variants in exon 3B of *PLP1* were detected: c.354_355delAG p.(G120PfsTer83), c.398A>C p.(H133P), c.435G>A p.(W145Ter) (Figure 1b,c). The variant was *de novo* in family 1; probands in family 2 and 3 inherited the mutation from the mother (aged 41 years in Family 2 and 36 years in Family 3). In family 3, also the maternal aunt (aged 35 years) and grandmother (aged 74 years) were tested positive for the [c.435 G>A; p.(W145Ter)]. All the female carriers have no neurological symptoms at their current ages.

## 4. Discussion

HEMS patients were first reported by Steenweg et al. in 2012 [4] and by Tonduti et al. in 2013 [5] based on a distinct hypomyelinating MRI pattern. Later in 2015, a total of nine variants, including point mutations in exon 3B or noncoding mutations in the adjacent intron 3 of the *PLP1*, were identified throughout exome sequencing in 16 male patients from 10 unrelated families [2] including cases reported for the first time [4,5]. Exonic mutations included synonymous, missense, and a small deletion (Figure 1c). Molecular studies and predictive tools suggested that variants in either exon 3B or intron 3 altered the PLP1/DM20 alternative splicing with subsequent reduction of the PLP1/DM20 ratio (i.e., decreased PLP1 expression in relation to DM20) [2]. We have expanded the mutational spectrum of HEMS-causing *PLP1* mutations by adding novel missense, nonsense, and frameshift variants in the exon 3B. Both the p.(G120PfsTer83) and p.(W145Ter) variants result in a premature stop codon of the PLP1, and thus they are likely to act as the p.(R127KfsTer16), that was predicted to truncate PLP1 but not DM20 [2]. The number of HEMS-causing mutations so far reported raises to twelve, seven of them being exonic and the remaining five intronic. In view of these data and following our experience, we recommend prioritizing the direct sequencing of exon 3B and intron 3 when HEMS is suspected, also because particularly intronic variants can be missed by the current use NGS custom panels and whole-exome sequencing. Although our three variants are novel, several other mutations in exon 3B have been described [6,7,8,9,10,11,12,13,14,15] and have been associated with either classic PMD [9,10,13,14], “mild” PMD [12,15], or SPG2 phenotype [6,7,8,11]. Most of these reports were published several years before the recognition of the HEMS neuroimaging pattern.

From a clinical point of view, the core phenotype of HEMS has been described as an ataxic, ataxic–spastic, or spastic syndrome, whose initial manifestations range from birth to 5 years. Cognition may not be affected or may be mildly-to-moderately impaired. Motor outcome is variable since about 40% of cases can walk unsupported, and the remaining cases need support or are not able to walk regardless of their age at onset or duration of the disease. Episodes of regression have been reported only in a minority of cases (i.e., 12.5%) [2]. Our series of patients confirm that the severity of HEMS phenotype is intermediate between the more severe connatal or classic PMD and the milder pure SPG2. Additionally, a certain degree of phenotypic variability exists in terms of syndromic presentation and severity of the disease. The onset of disease was detected in the first year of age, and psychomotor delay was the most common presentation. Interestingly, one of our cases also manifested orofacial dyskinesia from the age of 7. Extrapyramidal features are more frequently observed in severe *PLP1*-related phenotypes as connatal or classic PMD, but they have not been reported in HEMS [2]. It must also be considered that, although rare, episodes of motor worsening can occur in late adolescence, as observed in one of our cases.

Brain MRI confirmed the involvement of structures that physiologically myelinate early, specifically brainstem, PLIC, and optic radiations. Interestingly, brain MRI did not show any involvement of medulla, midbrain, and hilus of the dentate nuclei in the single case (i.e., patient 1) who had his first neuroradiological study performed after the age of 2. As reported in several HEMS cases by Kelavam and colleagues [2], improvement of myelination of some structures (e.g., medulla, pons, dentate nuclei) and worsening in others (e.g., periventricular white matter) have also been observed in patient 3. Consequently, these data suggest that the pattern of hypomyelination in HEMS may change over time and may be related to the age at which brain MRI is performed, but the involvement of some structures (i.e., PLIC, optic radiations) is mandatory and can be considered as the neuroradiological hallmark of the disease and generally does not change with time.

## 5. Conclusions

By reporting three new families harboring novel mutations in the exon 3B of the *PLP1*, we contribute to expanding the phenotypic and genotypic spectrum of HEMS. Although a limited number of patients have been described, it seems that this hypomyelinating leukodystrophy has well-defined clinical (i.e., the onset of psychomotor delay and/or nystagmus evolving into ataxic-spastic syndrome), genetic (i.e., mutations in the exon 3B or intron 3 of *PLP1*), and neuroradiological (i.e., involvement of early myelinated structures) features. Further studies, especially enriched by longitudinal functional evaluations and long-term follow-up, are need to better delineate the natural history of this rare *PLP1*-related disorder.

## Figures and Tables

**Table 1:** Main clinical features of reported patients.

Patient	1	2	3	4
General data				
*PLP1* mutation	c.354_355delAG; p.(G120PfsTer83)	c.398A>C; p.(H133P)	c.435G>A; p.(W145Ter)	c.435G>A; p.(W145Ter)
Male/Female	M	M	M	M
Ethnicity	Georgian	Italian	Italian	Italian
Current age	6 years 4 months	3 years 4 months	8 years	46 years
Familial/Isolated	I	I	F	F
Presentation				
Age at onset	8 months	6 months	9 months	6 months
Signs at onset	Psychomotor delay	Nystagmus	Psychomotor delay	Psychomotor delay
First examination				
Age	4 years 5 months	2 years	1 year	40 years
Eye movements	No	Nystagmus	Strabismus, nystagmus	No
Muscle tone	Mild axial and lower limbs hypotonia	Mild hypertonia	Axial hypotonia, limbs hypertonia	Limbs hypertonia
Pyramidal signs	Yes (mildly increased OTR)	Yes (increased OTR)	Yes (Babinski, clonus, increased OTR)	Yes (Babinski, clonus, increased OTR)
Extrapyramidal signs	No	No	Yes (orofacial dyskinesia)	No
Bulbar signs	No	No	Yes (dysphagia)	No
Cerebellar signs	Yes (ataxia, upper limbs tremor, dysarthria)	Yes (ataxia, mild dysmetria, and dysarthria)	Yes (head and trunk titubation, upper limbs tremor, dysarthria)	Yes (ataxia, upper limbs tremor, dysarthria)
Sensory function	Normal	Normal	Normal	Normal
Gait	Supported	Unsupported	Supported	Lost
Clinical course				
Age at last evaluation	5 years 6 months	3 years 1 month	8 years	46 years
Functional	Ataxic syndrome	Ataxic-spastic syndrome	Ataxic-spastic syndrome	Ataxic-spastic syndrome
Motor outcome	Sitting position 2 years; able to walk with light support from age of 4 years	Sitting position 1 year; autonomous walking from age of 2 years; running possible	Sitting position 3 years; able to walk with stroller from age of 6.5 years	Able to walk from 2.5 to 20 years
Cognitive outcome	Speak with simple sentences from age of 3 years	First words from 2 years; speak with complex sentences from age of 3 years	Limited speech, only few words easy to understand)	Unintelligible speech
Regression	No	No	No	Loss of ambulation
Other features	No	No	No	No
GMFCS	III	I	III	NA
EDACS	I	I	III	NA
MACS/Mini-MACS	II	II	III	NA
CFCS	II	I	III	NA
VSS	II	I	III	IV

Abbreviations: CFCS, Communication Function Classification System; EDACS, Eating and Drinking Ability Classification System; MACS, Manual Ability Classification System; NA, not available; OTR, osteotendinous reflexes; VSS, Viking speech scale; M, male; F, female.

**Table 2:** Detailed brain MRI characteristics.

Patient	1	2	3
Age at first MRI	4 years 4 months	2 years 1 months	1 years 9 months
Increased T2 signal			
Medulla	−	+	+
Pons	+ (mild, posterior)	+ (peripheral)	+ (posterior)
Midbrain	−	+	−
Dentate nuclei	−	+	+
Hilus of dentate nuclei	−	+	+
Cerebellar WM	−	−	−
Optic radiations	+	+	+
Periventricular WM	+ (very mild)	+	+
IC, anterior limb	−	−	−
IC, posteriori limb	+ (poor definition, stripes)	+ (tram track)	+ (tram track)
Corpus callosum	−	−	−
Subcortical WM	+ (very mild)	+	+
Basal ganglia	−	−	−
Thalami	−	+ (dorsomedial)	+ (dorsomedial)
Cerebral cortex	−	−	−
Age at last MRI	5 years 5 months	NP	7 years 8 months
Variations	No	NA	Yes
Improvement	−	NA	Medulla, DN, pons
Unchanged	−	NA	Optic radiations, PLIC, thalami
Worsening	−	NA	Periventricular and subcortical WM

List of abbreviations: DN, dentate nuclei; IC, internal capsulae; NP, not performed; NA, not applicable; PLIC, posterior limb internal capsulae; WM, white matter.

## Data Availability

The data presented in this study are available on reasonable request from the corresponding author.

## Supplementary Materials

The following are available online at https://www.mdpi.com/2076-3425/11/1/93/s1, Figure S1: Evolution of brain MRI anomalies in patient 3 (please refer to Table 2 for details). Table S1: Genotype-phenotype correlations in *PLP1*-related disorders.
