# Supplementary material for: Expanding the Clinical and Mutational Spectrum of the PLP1-Related Hypomyelination of Early Myelinated Structures (HEMS)
Source: Brain Sci. 2021 Jan 13;11(1):93. doi: 10.3390/brainsci11010093 (PMC7828325; doi:10.3390/brainsci11010093)

Supplementary Files

Table S1: Genotype-phenotype correlations in PLP1-related disorders, listed by increasing severity, from the most severe (i.e. connatal and classic PMD) to intermediate (i.e. PLP1-null syndrome, complex SPG2 and HEMS) to mildest (i.e., pure SPG2) forms. ° The term "null" refers to a pathogenic variant that results in either no mRNA, no protein, or a nonfunctional protein.

Phenotype	Genotype	Age of onset	Neurological features	Brain MRI	Disease's course
**Connatal PMD**	Missense (in highly conserved regions); rarely duplication (three or more copies)	Neonatal period	Nystagmus from birth; severe hypotonia evolving into spasticity; laryngeal stridor; dysphagia; seizures are possible	Severe diffuse hypomyelination	Severe intellectual disability with absent language; no autonomous walking; Age at death: infancy to 3th decade
**Classic PMD**	Duplication	1-5 year	Nystagmus in the first 1-2 months of life; hypotonia evolving into spasticity; ataxia head titubation; psychomotor delay; extrapyramidal features (dystonia, choreo-athetosis)	Diffuse hypomyelination	If acquired, deambulation is usually lost in infancy or in adolescence; verbal communication possible; Age at death: 3th-7th decade
***PLP1*-null syndrome**	"Null" mutation°	1-5 year	No nystagmus; mild spastic quadriparesis; ataxia; peripheral demyelinating neuropathy; psycho-motor delay	Diffuse hypomyelination	Mild-to-moderate cognitive impairment; Autonomous walking and verbal communication are present; Age at death: 5th-7th decade
**SPG2 (complex form)**	Missense (in less conserved regions)	1-5 year	Nystagmus; spastic-ataxia; autonomic dysfunction (spastic urinary bladder); mild psychomotor delay	Less severe abnormalities consisting in T2-weighted patchy abnormalities or more diffuse hypomyelination	Walking and verbal communication are present; Mild-to-moderate cognitive impairment Age at death: 4th-7th decade
**HEMS**	Intron or exone 3B	1-2 year	Nystagmus; spastic-ataxia; autonomic dysfunction (spastic urinary bladder); mild psychomotor delay	Hypomyelination of early myelinated structures (erebellum, brainstem, optic radiations, posterior limb of the internal capsulae)	Walking and verbal communication are present; Mild-to-moderate cognitive impairment
**SPG2 (pure form)**	Missense (in less conserved regions)	1-5 year; rarely 3th-4th decade	Spastic paraparesis with no adjunctive features	Less severe abnormalities consisting in T2-weighted patchy abnormalities	Walking and verbal communication are present; No cognitive delay

Figure S1. Evolution of brain MRI anomalies in patient 3 (please refer to Table 2 for details).

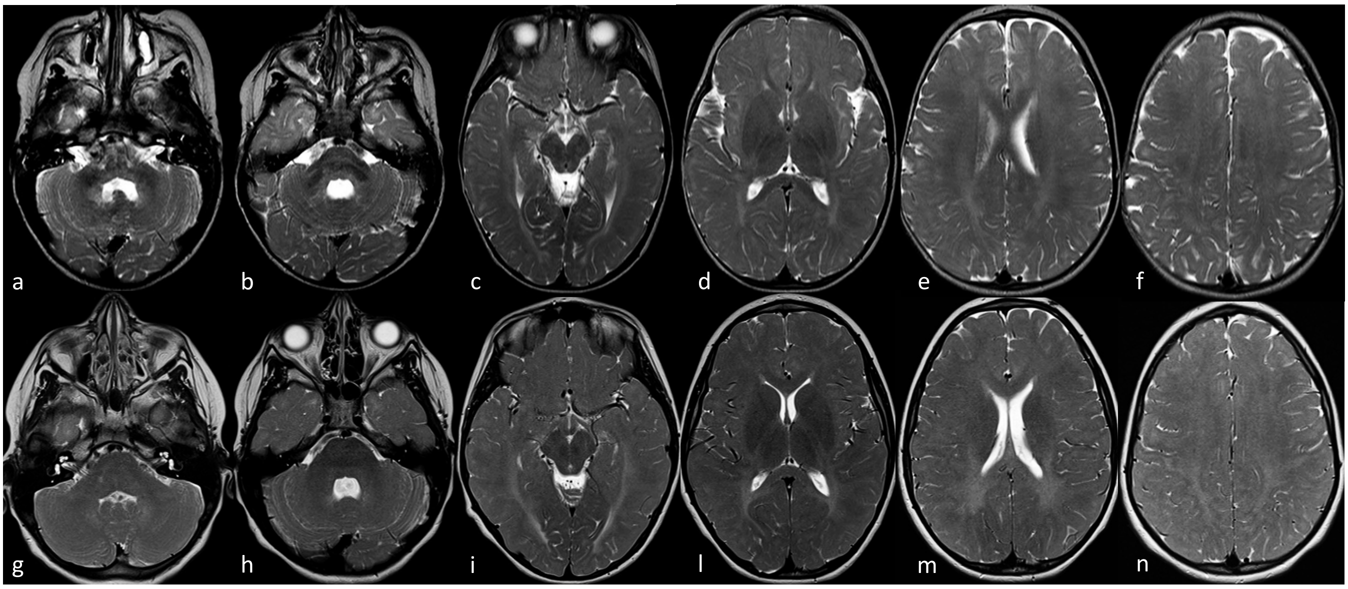

Supplement: Supplementary file 1 [file brainsci-11-00093-s001.pdf]
